# Possible Involvement of Nitric Oxide in Enhanced Liver Injury and Fibrogenesis during Cholestasis in Cytoglobin-deficient Mice

**DOI:** 10.1038/srep41888

**Published:** 2017-02-03

**Authors:** Tuong Thi Van Thuy, Le Thi Thanh Thuy, Katsutoshi Yoshizato, Norifumi Kawada

**Affiliations:** 1 Department of Hepatology, Graduate School of Medicine, Osaka City University, Osaka, Japan; 2 Synthetic Biology Laboratory, Graduate School of Medicine, Osaka City University, Osaka, Japan; 3 PhoenixBio Co. Ltd., Hiroshima, Japan

## Abstract

This study clarified the role of Cygb, the fourth globin in mammals originally discovered in rat hepatic stellate cells (HSCs), in cholestatic liver disease. Bile duct ligation (BDL) augmented inflammatory reactions as revealed by increased infiltrating neutrophils, CD68^+^-macrophages, and chemokine expression in Cygb^−/−^ mice. In these mice, impairment of bile canalicular indicated by the loss of CD10 expression, down-regulation of bile salt transporters, increased total bile acid, and massive apoptotic and necrotic hepatocytes occurred with the release of cytochrome c, activation of caspase 3, resulting in reduced animal survival compared to wild-type mice. In Cygb^−/−^ mouse liver, all of NO metabolites and oxidative stress were increased. Treatment with NO inhibitor restrained all above phenotypes and restored CD10 expression in BDL Cygb^−/−^ mice, while administration of NO donor aggravated liver damage in BDL-wild type mice to the same extent of BDL-Cygb^−/−^ mice. N-acetylcysteine administration had a negligible effect in all groups. In mice of BDL for 1–3 weeks, expression of all fibrosis-related markers was significantly increased in Cygb^−/−^ mice compared with wild-type mice. Thus, Cygb deficiency in HSCs enhances hepatocyte damage and inflammation in early phase and fibrosis development in late phase in mice subjected to BDL, presumably via altered NO metabolism.

Cholestatic liver disease is caused by the dysregulated production and excretion of bile from the liver to duodenum, which induces jaundice and the injury of the bile duct and hepatocytes, leading to biliary fibrosis, cirrhosis, and liver failure if persisted^1^. Uncovering the pathophysiology under of cholestatic disorders may be challenging for the development of therapeutic approaches to human cholestatic liver diseases. A well-established model of obstructive jaundice in mice that mimics human disease is bile duct ligation (BDL)^2^. To date, mechanisms involved in BDL-induced liver injuries were reported to include three inflammatory phenotypes^2^,^3^,^4^: (1) an acute phenotype characterized by a hepatocellular injury phase induced by the accumulation of excessive hydrophobic bile acid; (2) a sub-acute phenotype, namely the leukocytic phase, in which activated neutrophils infiltrate and attack the toxic bile acid-stressed hepatocytes through excessive reactive oxygen species (ROS); (3) a chronic phenotype, namely the angiogenic phase, wherein new vessels are formed around biliary tracts for oxygen supply and antioxidant and anti-immune properties.

Cytoglobin (Cygb) was originally identified in 2001 as a protein expressed in rat hepatic stellate cells (HSCs)^5^. Cygb is expressed ubiquitously in the cytoplasm of pericytes in many organs, including the brain, thymus, heart, lung, liver, kidney, small intestine and spleen^6^. Functions of Cygb are supposed to include (1) O_2_ storage, diffusion and sensing for cellular respiration and metabolism^5^,^7^, (2) nitric oxide (NO) scavenging^8^,^9^, and (3) involvement in hypoxia and oxidative stress^10^. Indeed, the NO dioxygenase (NOD) activity of Cygb is one of the most studied issues to date. Smagghe and colleagues examined the NOD activity of various globins in their oxy-ferrous state, and Cygb exhibited the highest consumption rate^11^. At low O_2_ levels (0–50 mM), Cygb and other cellular reductants regulated the rate of NO consumption in a manner dependent on O_2_ concentration, showing ~500-fold greater sensitivity to changes in O_2_ level than myoglobin (Mb)^12^. On the other hand, Gardner *et al*. reported that the NO-scavenging function of Cygb protected the NO-sensitive aconitase, decrease peroxynitrite (ONOO^−^) formation and protected cellular respiration in rat hepatocytes^8^. In general, the accumulation of ONOO^−^ and other nitrosative molecules affect the interactions with lipids, DNA, and proteins via direct oxidative reactions and nitration or via indirect, radical-mediated mechanism^13^. Thus, the NO scavenging function of Cygb seems to be crucial for protecting cells and tissues from NO toxicity.

Given the implication of Cygb in numerous vital functions, we generated Cygb-deficient (Cygb^−/−^) mice^14^ and reported their high susceptibility to tumour development in the liver and lungs when treated with *N, N*-diethylnitrosamine (DEN)^14^. Furthermore, Cygb^−/−^ mice exhibited augmented inflammation, fibrosis and cancer development in a non-alcoholic steatohepatitis (NASH) model induced by a choline-deficient L-amino acid-defined diet via activation of the oxidative stress pathway^15^. Cygb^−/−^ mice ranging from 1 to 2 years of age spontaneously displayed multiple organ abnormalities, including heart hypertrophy and tumours in the lung, liver, ovary, small intestine and lymphatic organs^16^. These findings suggest that Cygb may be an important protector of all organs, especially in the liver.

Here, we described the exacerbation of hepatocyte death, hepatic inflammation and fibrogenesis following BDL in Cygb deficiency. The possible involvement of NO in the pathogenesis will be discussed.

## Results

### Cygb deficiency aggravated liver injury following BDL

**Figure 1:**
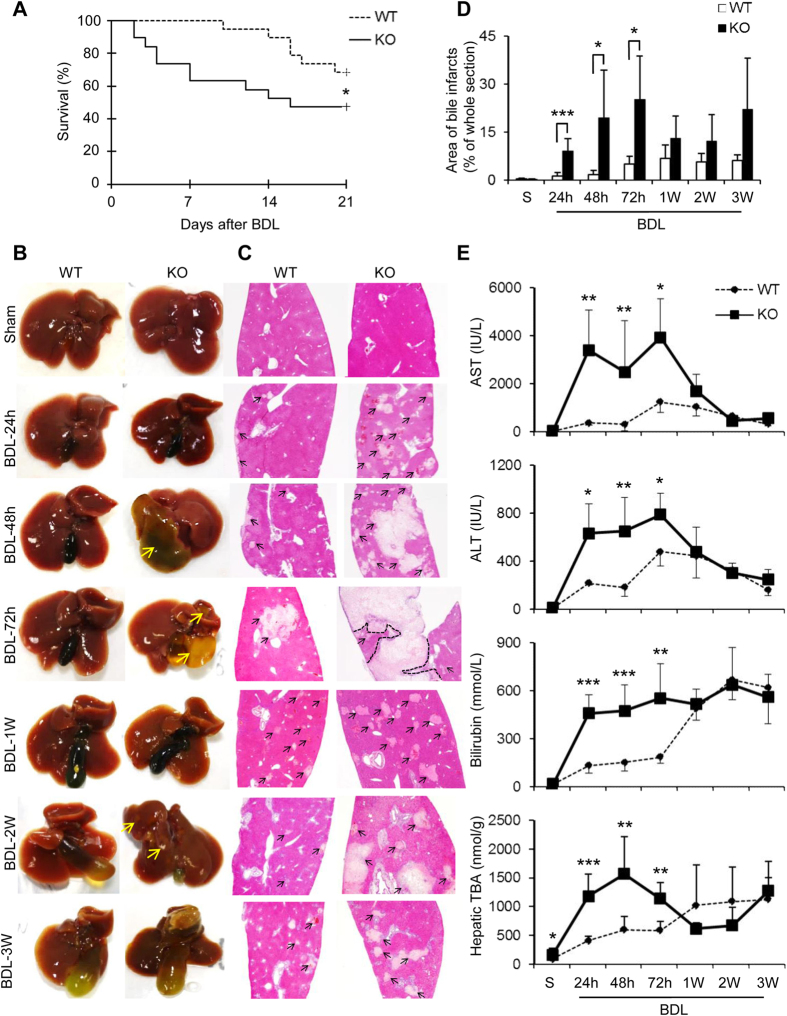
Severe liver injury in Cygb^−/−^ mice under BDL. (**A**) Kaplan-Meier curve, n = 19 per group. Representative macroscopic images (**B**) and microscopic liver sections stained with H&E (**C**) in sham, acute BDL (24–72 h) and chronic BDL (1–3 W). Original magnification, x40. Yellow and black arrows indicate bile infarcts. (**D**) Quantification of area of bile infarcts. (**E**) Levels of serum AST, ALT, and total bilirubin, and hepatic total bile acid (TBA). Data represent the mean ± SD. Sham (n = 3), BDL (n = 4–8). *p < 0.05, **p < 0.01, ***p < 0.001.

BDL was employed to induce mechanical blockage of the bile duct system in wild-type (WT) and Cygb^−/−^ mice. The deficiency of Cygb was confirmed in Cygb^−/−^ mice (Supplementary Fig. S1A–D). Cygb exists in HSCs but not in hepatocytes^15^ or other inflammatory cells in the liver (Supplementary Fig. S1E). The survival rate revealed that 7 out of 19 (37%) Cygb^−/−^ mice were died at 7 days after BDL whereas all WT mice were still alive (Fig. 1A). Thereafter, at day 21, the survival rate was 47% in Cygb^−/−^ mice, which was significantly different from WT (68%) (Fig. 1A). Thus, BDL significantly reduced the survival rate in Cygb^−/−^ mice (p < 0.05).

At a macroscopic view, BDL induced the time-dependent widening of gall bladders and a minor irregularity of liver surfaces in WT mice (Fig. 1B). In contrast, BDL-Cygb^−/−^ livers exhibited large green-yellowish areas in both acute (48–72 h) and chronic phase (2–3 weeks) (Fig. 1B). Hematoxylin and eosin (H&E) staining exhibited the presence of multi and large foci of parenchymal degeneration in Cygb^−/−^ livers, namely bile infarcts, as early as 24 h after BDL (Fig. 1C) compared with a few small ones in WT. The area of bile infarct was increased in BDL-Cygb^−/−^ mice compared with in BDL-WT at all time-points (Fig. 1D).

Serum levels of alanine transaminase (AST), aspartate transaminase (ALT) and total bilirubin (T-Bil) and hepatic total bile acid (TBA) in BDL-Cygb^−/−^ mice were markedly increased by 3- to 10-fold at 24 h, and then peaked at 48 or 72 h compared with WT (Fig. 1E). TBA, a marker of cholestasis, peaked at 48 h after BDL both in serum and liver in Cygb^−/−^ mice compared with that at 1 week in serum and at 3 week in liver in WT (Fig. 1E and Supplementary Fig. S2A). These results were derived from the obstructive bile ducts, but not from the synthesis pathway, as indicated by the down-regulated transcription levels of cytochromes P450 7A1 (Cyp7a1) and P450 7B1 (Cyp7b1), two main enzymes involved in the bile acid synthesis (Supplementary Fig. S2B). Altogether, the loss of Cygb aggravated BDL-induced liver damage in mice.

### Cygb deficiency promoted hepatic inflammation and cell death under BDL

**Figure 2:**
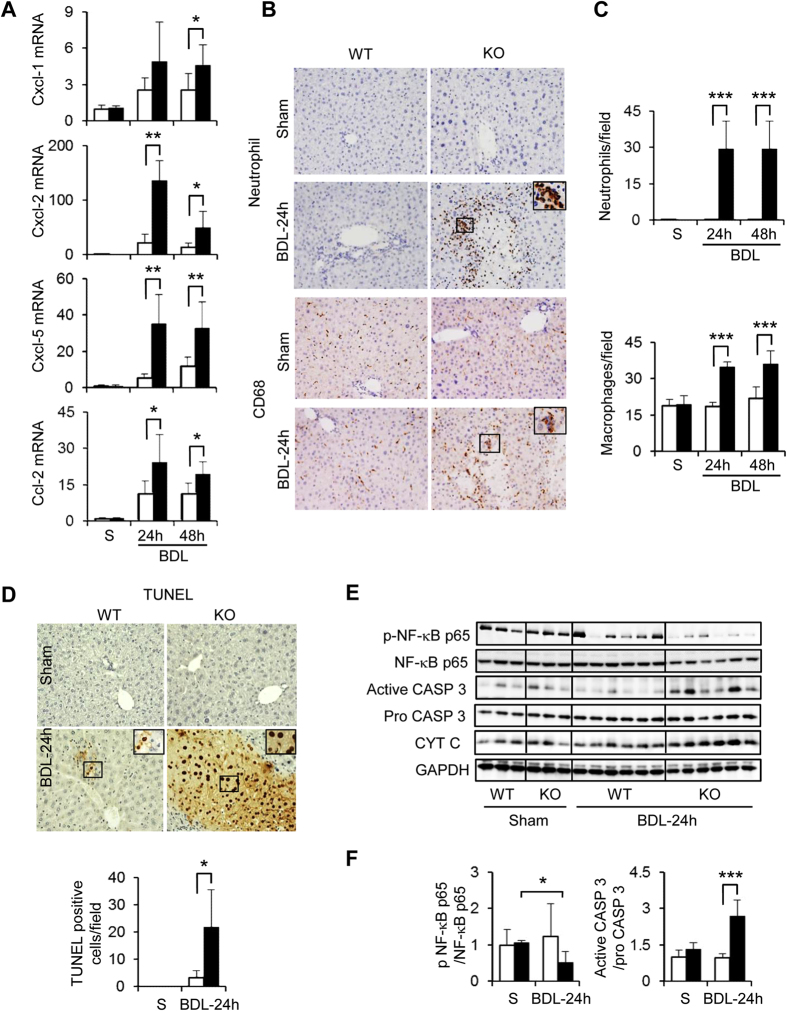
Effect of Cygb deficiency on inflammation and cell death in acute BDL. (**A**) Hepatic mRNA level of chemokine Cxcl-1, Cxcl-2, Cxcl-5, and Ccl-2 in sham (S) and acute BDL (24–48 h). (**B**) Immunohistochemistry of neutrophil- (top panels) and CD68- positive cells (bottom panels) in sham and BDL-24 h mice. (**C**) Quantification of neutrophil- (top panel) and CD68-positive cells (bottom panel) per field. (**D**) TUNEL staining in sham and acute BDL-24 (top panels) and number of TUNEL positive cells (bottom panel) per field. (**E**) Immunoblots of phosphorylated (p) and total NF-κB p65, active and pro caspase 3 (CASP 3), and cytochrome c (CYT C) in sham and BDL-24 h mice. GAPDH was used as loading control. All gels were run under the same experimental conditions. The cropped gels are used and full-length gels are presented in Supplementary Fig. S8. (**E**) Quantitative densitometry of p-NF-κB and active CASP 3 in sham and BDL-24 h. Open bars, WT; close bars, Cygb^−/−^. Data represent the mean ± SD. Sham (n = 3), BDL (n = 4–8). *p < 0.05, **p < 0.01, ***p < 0.001. Original magnification, x400; inset, x1200.

One important feature of obstructive cholestasis is hepatic inflammation with neutrophil infiltration and the activation of resident and infiltrated macrophages^2^. First, we analysed the hepatic mRNA levels of chemokines that have a role in initiating inflammation and found that Cxcl1, Cxcl2, Cxcl5, and Ccl2 were up-regulated in Cygb^−/−^ mice compared with WT in the acute phase of BDL (Fig. 2A). In addition, the number of neutrophils and CD68^+^ macrophages increased instantly in Cygb^−/−^ mouse liver after BDL (Fig. 2B,C).

To determine which mechanism induced the aggravated liver injury in Cygb^−/−^ mice, we evaluated dead hepatocytes by terminal deoxynucleotidyl transferase–mediated deoxyuridine triphosphate nick-end labeling (TUNEL) assay which detects both apoptotic and necrotic cells. Hepatocytes of Cygb^−/−^ mice exhibited aberrant TUNEL-positive cells compared with WT (Fig. 2D). High concentrations of bile acids increase mitochondrial permeability^3^,^17^ which was demonstrated to be involved in both apoptotic and necrotic cell death^18^,^19^. An increase in the permeability of the outer mitochondrial membrane is crucial for apoptosis with the release of several apoptogenic factors into cytoplasm, such as cytochrome (Cyt) c. On the other hand, an increase in the permeability of both the outer and inner mitochondrial membranes leads to necrosis^19^. Here we found the re-localization of Cyt c from the mitochondria to the cytosol around the bile infarcts, which was accompanied by caspase (Casp) 3 activation in Cygb^−/−^ liver, as early as 24 h after BDL with levels greater than those in WT mice (Fig. 2E,F and Supplementary Fig. S3A). However, hepatic Casp 3 activity in BDL-Cygb^−/−^ was not significantly increased compared with WT-BDL mice (Supplementary Fig. S3B). NF-κB, which is activated in cholestasis and functions as promoting survival gene expression^20^, was inhibited in Cygb^−/−^ mice as demonstrated by decreased p-NF-κB p65 expression compared with WT (Fig. 2E,F). Taken together, the loss of Cygb induced huge clusters of apoptotic and necrotic hepatocytes under BDL.

### Cygb deficiency enhanced the impairment of bile canaliculi and the down-regulation of both canalicular and basolateral bile transporters in hepatocytes under BDL

**Figure 3:**
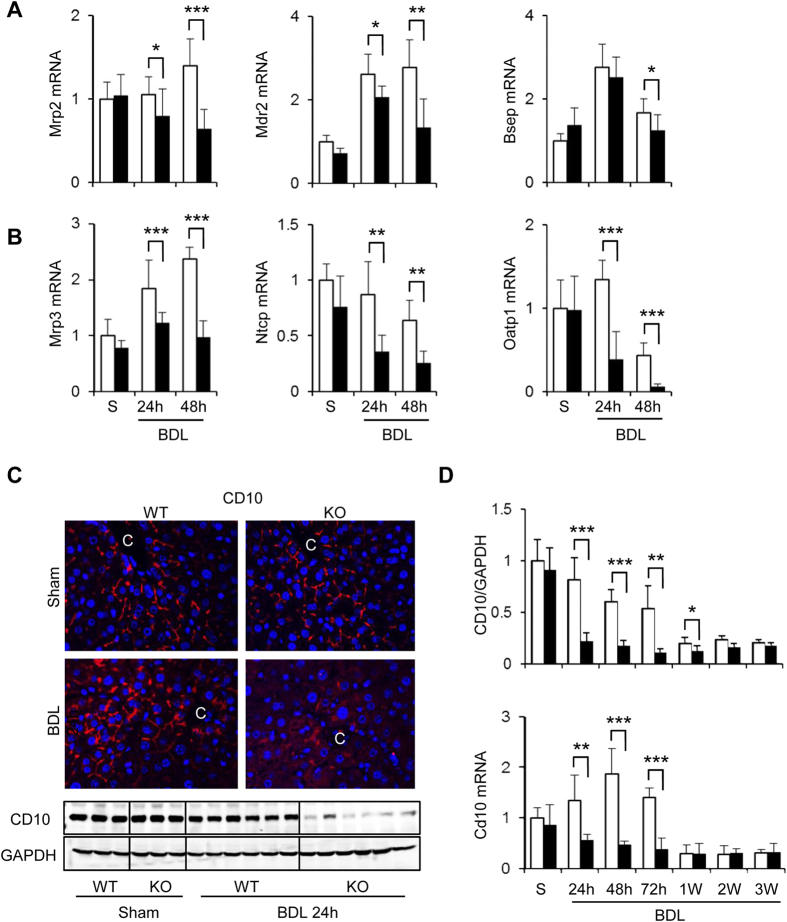
Effect of Cygb deficiency in the expression of bile transporters and CD10 in acute BDL mice. Hepatic mRNA level of (**A**) sinusoidal (Mrp2, Mdr2, Bsep) and (**B**) canalicular (Mrp3, Ntcp, Oatp1) transporters of bile components in sham (S) and acute BDL (24–48 h) mice. (**C**) Immunofluorescence (top panels) and immunoblot (bottom panels) of CD10 in sham and BDL-24 h mice. GAPDH was used as loading control. All gels were run under the same experimental conditions. The cropped gels are used and full-length gels are presented in Supplementary Fig. S9. (**D**) Quantitative densitometry of CD10 (top panel) and hepatic mRNA level (bottom panel) of CD10 in sham (S) and acute BDL (24–72 h) and chronic BDL (1–3 W) mice. Open bars, WT; close bars, Cygb^−/−^. Data represent the mean ± SD. Sham (n = 3), BDL (n = 4–8). *p < 0.05, **p < 0.01, ***p < 0.001. Original magnification, x400.

Because the adaptive regulation of membrane transporters limits hepatotoxicity in obstructive cholestasis^21^, we determined the mRNA levels of three main hepatic efflux transporters multidrug resistance-associated protein (Mrp) 2, multidrug resistance P-glycoprotein (Mdr) 2, and bile salt export pump (Bsep) in the canalicular membrane and found that the expression of these transporters was significantly down-regulated in BDL-Cygb^−/−^ liver compared with BDL-WT livers (Fig. 3A). Moreover, the other hepatic efflux transporter, Mrp3, and hepatic uptake transporters, sodium taurocholate co-transporting polypeptide (Ntcp) and organic anion polypeptide transporter (Oatp) 1 in the basolateral membrane were also transcriptionally down-regulated in the BDL-Cygb^−/−^ liver (Fig. 3B).

Given that bile obstruction induces the enlargement of the lumina of the bile canaliculi and the disappearance of microvilli of bile canalicular membrane^22^, we next assessed the expression of CD10, an endopeptidase located on the microvilli^23^. After BDL, CD10 protein and mRNA levels were decreased in a time-dependent fashion and almost undetectable after 1 week in WT, whereas it was immediately attenuated at 24 h in Cygb^−/−^ liver (Fig. 3C,D, and Supplementary Fig. S4). Altogether, Cygb deficiency induced a more severe impairment of canalicular and sinusoidal transporters and membranes of hepatocytes, resulting in the deterioration of hepatocyte damage.

### Cygb deficiency augmented nitrosative and oxidative stress under BDL

**Figure 4:**
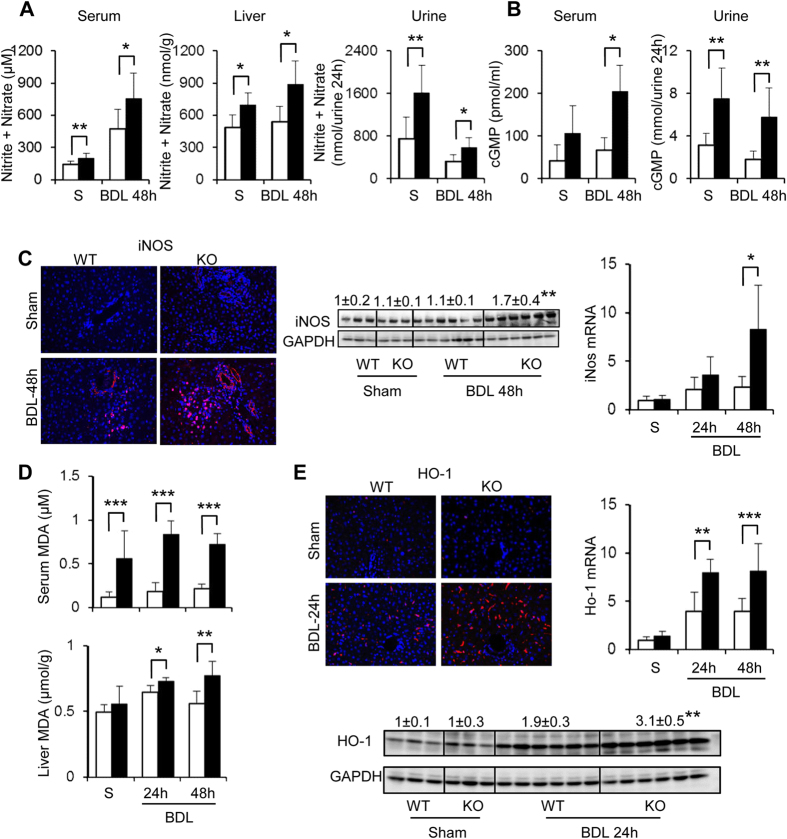
NO metabolites and oxidative stress condition in Cygb^−/−^ mice under acute BDL. Concentration of nitrite + nitrate (**A**) and cGMP (**B**) in serum, liver lysate and urine in sham (S) and acute BDL-48 h mice. (**C**) Hepatic immunofluorescence, immunoblot and mRNA level of iNOS in sham and acute BDL (24–48 h) mice. (**D**) Malondialdehyde (MDA) content of sham (S) and acute BDL (24–48 h) mice in serum and liver. (**E**) Hepatic immunofluorescence, immunoblot and mRNA of HO-1 in sham or acute BDL mice (24–48 h). GAPDH was used as loading control. All gels were run under the same experimental conditions. The cropped gels are used and full-length gels are presented in Supplementary Fig. S10. Open bars, WT; close bars, Cygb^−/−^. Data represent the mean ± SD. Sham (n = 3), BDL (n = 4–8). *p < 0.05, **p < 0.01, ***p < 0.001. Original magnification, x400.

Because Cygb scavenges NO and the other ROS^24^, we speculated that Cygb^−/−^ livers might suffer from toxicities by these molecules under BDL. We first assessed the concentration of nitrite + nitrate, the oxidized products of NO, in serum, liver and urine and guanosine 3′,5′-cyclic monophosphate (cGMP) level in serum and urine. All of these molecules were significantly increased in Cygb^−/−^ mice compared with WT mice in BDL 48 h (Fig. 4A,B) and subject to sham operation, indicating that Cygb^−/−^ mice suffered from NO toxicity^16^. The elevation of NO in BDL-Cygb^−/−^ mice might reflect not only the loss of NO scavenging function of Cygb but also from the increased NO production in inflammatory conditions of Cygb^−/−^ livers. Indeed, the high expression of inducible nitric oxide synthase (iNOS) at both mRNA and protein levels in BDL-Cygb^−/−^ livers compared with WT mice was demonstrated at 48 h (Fig. 4C). These results suggest that the loss of Cygb may promote BDL-induced liver injuries presumably through the augmentation of NO toxicity in Cygb^−/−^ mice.

With regard to ROS production, we measured the level of malondialdehyde (MDA), an end product of lipid peroxidation, and found that it was significantly elevated in both serum and liver tissue in BDL -Cygb^−/−^ mice compared with WT mice (Fig. 4D). Consistent with our previous study^16^, the high levels of MDA were also evident in sham operated Cygb^−/−^ mice, indicating the spontaneous oxidative stress condition in Cygb^−/−^ mice. Furthermore, heme oxygenase-1 (HO-1), which is identical to heat shock protein 32 (HSP32) and is another popular oxidative stress marker, was markedly induced in BDL-Cygb^−/−^ mice liver compared with BDL-WT (Fig. 4E). Thus, the loss of Cygb dysregulated the production of NO and ROS that might consequently aggravate liver injuries in BDL-Cygb^−/−^ mice.

### Involvement of Nitric oxide in Liver Damage under BDL

**Figure 5:**
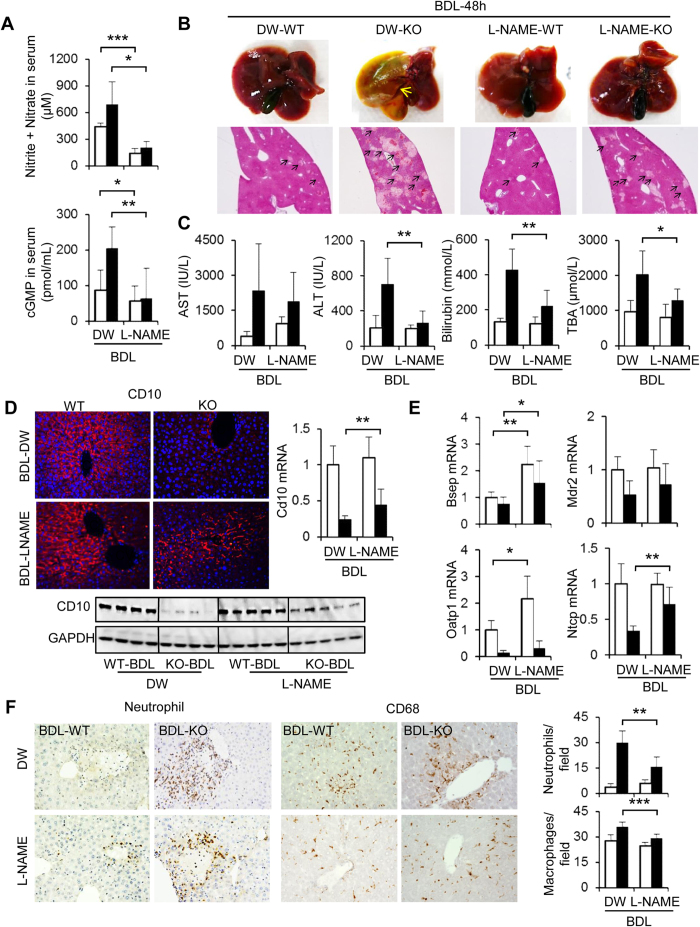
Effect of NO inhibitor in Cygb^−/−^ mice after BDL. WT and KO mice were subjected to BDL-48 h together with L-NG-nitroarginine methyl ester (L-NAME) treatment. Control mice received drinking water (DW). (**A**) Concentration of nitrite + nitrate and cGMP in serum. (**B**) Representative macroscopic images and microscopic liver sections stained with H&E. (**C**) Serum levels of AST, ALT, total bilirubin, and total bile acid (TBA). (**D**) Immunofluorescence, immunoblots and hepatic mRNA level of CD10. GAPDH was used as loading control. All gels were run under the same experimental conditions. The cropped gels are used and full-length gels are presented in Supplementary Fig. S11. (**E**) Hepatic mRNA level of Bsep, Mdr2, Oatp1, Ntcp. (**F**) Immunohistochemistry of neutrophils and CD68 and its quantitative analyses (right insets). Open bars, WT; close bars, KO. Data represent the mean ± SD. n = 5. *p < 0.05, **p < 0.01, ***p < 0.001. Original magnification, x40 (H&E; B), x400 (CD10, neutrophil and CD68; **D** and **E**).

**Figure 6:**
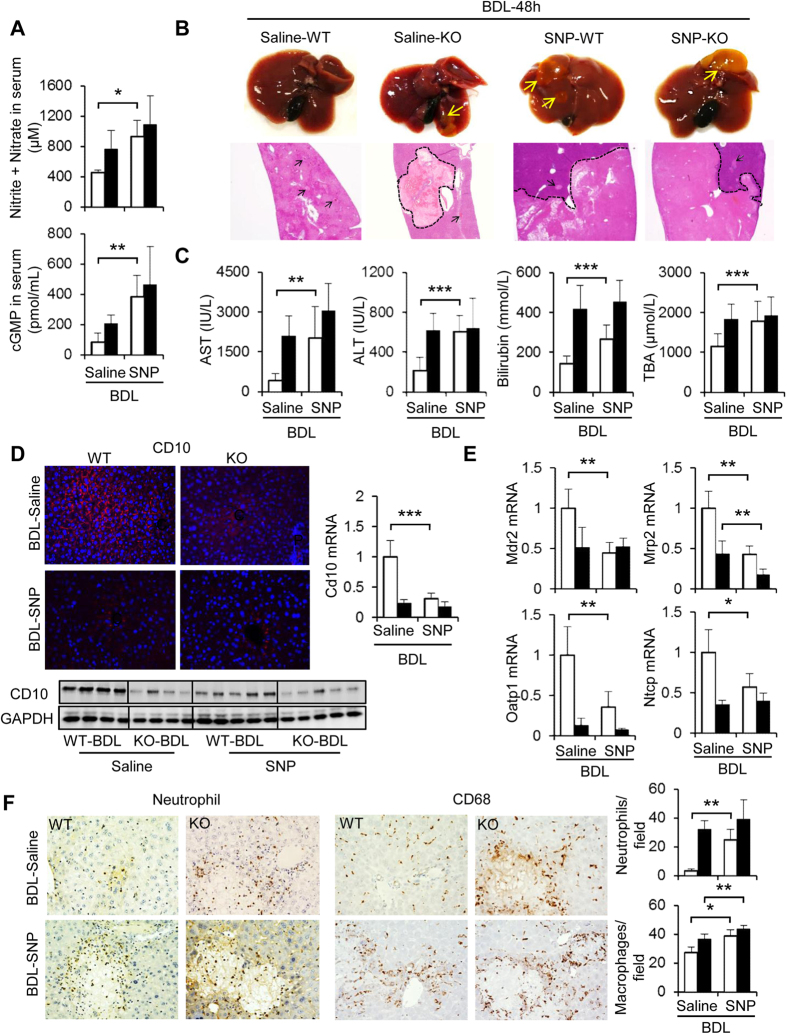
Effect of NO donor on BDL-induced liver injury in WT and Cygb^−/−^ mice after BDL. WT and KO were subjected to BDL-48 h together with saline or sodium nitroprusside (SNP) treatment. (**A**) Concentration of nitrite + nitrate and cGMP in serum. (**B**) Representative macroscopic images, microscopic liver sections stained with H&E. (**C**) Serum AST, ALT, total bilirubin, and total bile acid (TBA). (**D**) Immunofluorescent staining, immunoblots and hepatic mRNA level of CD10. GAPDH was used as loading control. All gels were run under the same experimental conditions. The cropped gels are used and full-length gels are presented in Supplementary Fig. S12. (**E**) Hepatic mRNA level of Bsep, Mdr2, Oatp1, Ntcp. (**F**) Immunohistochemistry of neutrophils and CD68 and its quantitative analyses (right insets). Open bars, WT; close bars, KO. Data represent the mean ± SD. n = 5. *p < 0.05, **p < 0.01, ***p < 0.001. Original magnifications, x40 (H&E; **B**); x400 (CD10, neutrophil and CD68; **D**,**E**).

To ascertain the role of NO in BDL-induced liver injuries in Cygb^−/−^ mice, we next studied the effect of an NO inhibitor or NO donor. BDL for 48 h, the point at which the maximum liver damage occurred in Cygb^−/−^ mice, was chosen for these experiments. The administration of L-NG-nitroarginine methyl ester (L-NAME) (1) reduced the levels of NO metabolites and cGMP (Fig. 5A), (2) diminished BDL-induced biliary infarcts (Fig. 5B and Supplementary Fig. S5A), (3) significantly reduced serum levels of ALT, bilirubin and TBA (Fig. 5C), (4) maintained CD10 protein and mRNA expression (Fig. 5D), (5) significantly up-regulated the mRNA levels of bile transporters Bsep and Ntcp (Fig. 5E), and (6) obviously decreased the number of neutrophils and CD68^+^-macrophages (Fig. 5F) in BDL-Cygb^−/−^ liver.

In contrast, an NO donor, sodium nitroprusside (SNP), treatment for 48 h together with BDL obviously increased NO metabolites and cGMP in the serum (Fig. 6A) and exaggerated bile infarcts in BDL-WT livers to the equivalent level observed in BDL-Cygb^−/−^ livers (Fig. 6B and Supplementary Fig. S5B). Serum levels of AST, ALT, T-Bil, and TBA all significantly increased in BDL-WT livers under SNP treatment (Fig. 6C). Impressively, SNP administration significantly attenuated CD10 mRNA and protein level (Fig. 6D), reduced mRNA expression of bile transporters (Fig. 6E), and promoted the accumulation of neutrophils and CD68^+^-macrophages (Fig. 6F) in BDL-WT livers, and these effects were similar to BDL-Cygb^−/−^ livers without SNP. These results indicated that the administration of SNP aggravated liver injury in BDL-WT livers to the extent observed in BDL-Cygb^−/−^ livers.

### Effect of N-acetylcysteine in liver injury under BDL

We next investigated whether N-acetylcysteine (NAC), a well-known antioxidant, rescues acute BDL in Cygb^−/−^ mice. Although NAC administration improved BDL-induced MDA formation in both WT and Cygb^−/−^ groups (Supplementary Fig. S6A), unexpectedly, it had negligible effect in BDL-induced liver injuries (Supplementary Fig. S6B,C).

### Cygb deficiency promoted hepatic fibrosis under long-term BDL

**Figure 7:**
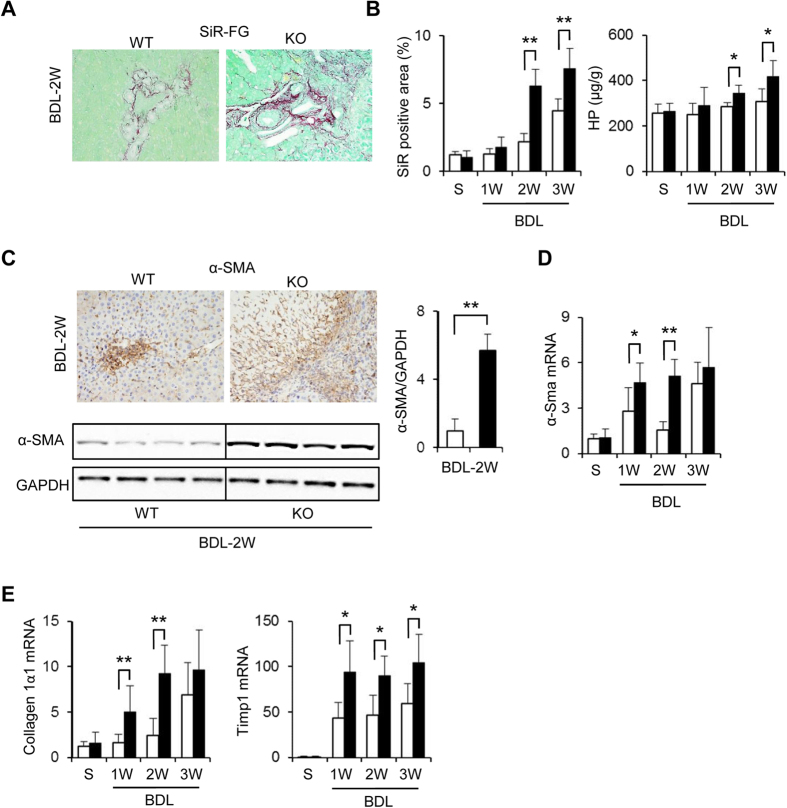
Promotion of fibrosis in Cygb^−/−^ mice after chronic BDL. (**A**) Liver sections from Sirius Red and Fast Green (SiR-FG) staining in BDL 2 W. (**B**) Sirius Red positive area (left panel) and hydroxyproline (HP) content of liver (right panel) in sham (S) and chronic BDL (1–3 W) mice. (**C**) Immunohistochemistry for α-SMA (top panels), immunoblot analysis (bottom panels), and its quantitative densitometry (right inset) of the α-SMA expression. GAPDH was used as loading control. All gels were run under the same experimental conditions. The cropped gels are used and full-length gels are presented in Supplementary Fig. S13. (**D**) Hepatic mRNA level of α-Sma expression. (**E**) Hepatic mRNA level of collagen1a1 and Timp-1. Open bars, WT; close bars, KO. Data represent the mean ± SD. Sham (n = 3), BDL (n = 4–8). *p < 0.05, **p < 0.01. Original magnification, x400.

Finally, we investigated the effect of Cygb deficiency on cholestasis-induced liver fibrosis. Sirius red and fast-green (SiR-FG) staining and hydroxyproline (HP) assay showed severe liver fibrosis development at week 2 and 3 in BDL-Cygb^−/−^ mice compared with WT (Fig. 7A,B). Because the activation of HSCs is the key factor in the development of hepatic fibrosis, αsmooth muscle actin (α-SMA), a marker of activated HSCs, was assessed and exhibited a significant increase in both protein and mRNA levels in Cygb^−/−^ mice not only in the chronic phase (Fig. 7C,D) but also from the acute phase (Supplementary Fig. S7). mRNA expression of other key genes involved in hepatic fibrogenesis, including collagen-1α1 and tissue inhibitor of matrix metalloproteinase-1 (Timp-1), was also up-regulated in Cygb^−/−^ compared with WT livers (Fig. 7E). These results demonstrated that the absence of Cygb augmented fibrosis development in the chronic phase under BDL.

## Discussion

### Loss of Cygb in HSCs aggravates hepatocyte damage under BDL by dysregulation of NO

Cygb is expressed in pericytes but not in the epithelial cells of all organs^6^. However, interestingly, the absence of Cygb promoted multiple organ abnormalities including tumours in the liver, lung, intestine, ovary, and lymphoid tissues, and heart hypertrophy and kidney fibrosis in mice^16^. Thus, the absence of Cygb gives rise to the whole body influence and dramatic liver injuries as shown in this BDL model. However, it is still unknown which factor linked to the absence of Cygb is crucial to induce these manifestations. Here we found that the dysregulation of NO metabolism based on Cygb deficiency might be responsible.

In the liver, NO plays important and diverse roles and can be both cytoprotective and cytotoxic^25^. A small amount of NO is crucial to maintain microcirculation, inhibit adhesion or emigration of neutrophils and aggregation of platelets^26^ or block cellular apoptosis by inhibiting caspase-3-like activity^27^. At high level, however, NO induces cellular apoptosis by modulating both extrinsic and intrinsic signalling pathways in Jurkat cells, freshly isolated human leukemic lymphocytes^28^ and rat hepatocytes^29^. This apoptosis-promoting effect of NO is likely associated with massive hepatocyte death in BDL- Cygb^−/−^ mice. Moreover, NO decreases the amount of NTCP in the hepatocyte plasma membrane via S-nitrosylation, resulting in the attenuation of NCTP-dependent uptake of bile acid into hepatocytes^30^. Consistent with this report, Ntcp mRNA levels were markedly decreased in BDL-Cygb^−/−^ mice (Fig. 3B). Importantly, we have shown the prompt and marked increase of TBA in the liver and serum of Cygb^−/−^ mice which may result in more bile infarcts than WT mice. While the bile acid synthesis-related genes, such as Cyp7a1 and Cyp7b1, were down-regulated (Supplementary Fig. S2B), the increased level of TBA in Cygb^−/−^ mice should be resulted from the obstruction of common bile duct which gave rise to the reflux of bile flow to the bile canaliculi. Jean-Francois *et al*. indicated that increased NO concentration blocked bile canalicular contraction in rat hepatocyte doublets^28^. Thus, the impairment of bile canaliculi, indicated by the loss of CD10, caused by high NO level in Cygb^−/−^ mice may induce the early leakage of bile flow into hepatocytes, resulting in marked hepatocyte death. Taken together, dysregulated NO metabolism due to the loss of Cygb induced explosive bile infarcts and decreased the survival rate in BDL-Cygb^−/−^ mice.

### Regulation of NO reverses phenotype of liver injury in BDL mice

Administration of L-NAME, a non-selective inhibitor of NOS, suppressed liver injury in BDL-Cygb^−/−^ mice (Fig. 5B,C and Supplementary Fig. S5A). Similarly, the decrease of NO production by the iNOS-specific inhibitor, L-N6-(1-iminoethyl)-lysine and S-methylisothiourea sulphate reduces hepatocellular necrosis in carbon tetrachloride-treated mice^31^. L-NAME also improves both structural abnormalities and apoptotic conditions in cardiac cells exhibiting cholestasis^32^. However, L-NAME administration did not thoroughly prevent the basal liver damage in both BDL-WT and Cygb^−/−^ mice at 48 h (Fig. 5B,C). It is plausible to hypothesize that liver injury at BDL-48 h occurred via multiple factors, especially toxic bile acids, and not exclusively via a NO-dependent mechanism in both WT and Cygb^−/−^ mice.

In contrast, when receiving SNP, severe hepatocyte injury occurred in BDL-WT mice similarly to BDL-Cygb^−/−^ mice (Fig. 6 and Supplementary Fig. S5B). Consistent with our study, Mayoral P. *et al*. reported that fibrogenesis was augmented dramatically in rats treated with L-arginine, a NO donor, under chronic BDL^33^. Although the liver injury in SNP-treated BDL-Cygb^−/−^ mice tended to be more severe than that of only BDL, the differences were not significant. It is hypothesized that BDL-induced liver trauma in Cygb^−/−^ mice reached the maximum extent, and SNP-treatment was unable to aggravate more the injury in BDL-Cygb^−/−^ mice at 48 h.

### Loss of Cygb augments inflammation and oxidative stress under BDL

In cholestatic liver disease, bile acids are inflammagens that stimulate hepatocytes to produce proinflammatory mediators, promoting the accumulation of neutrophils and other immune cells^34^. Accordingly, excessive bile acids accumulated in Cygb^−/−^ liver may explain the up-regulated expression of chemokines, resulting in the prompt and numerous recruitment of neutrophils and CD68^+^-macrophages 24 h after BDL compared with WT mice (Fig. 2B,C). These inflammatory cells are important sources of NO and the other superoxides that induce severe cholestatic liver injury.

Our previous studies showed that the robust accumulation of ROS was associated with the absence of Cygb under either diet-induced^15^ or chemical-induced^14^ liver injuries. In this cholestatic model, consistent with aberrant hepatocellular injury and numerous numbers of infiltrated neutrophils and macrophages, oxidative stress via MDA and HO-1 (Fig. 4D,E) was markedly elevated in Cygb^−/−^ mouse liver compared with WT. However, although NAC-administration reduced serum and hepatic MDA levels, it had negligible effect on liver injury, at least in acute phase in BDL-WT and Cygb^−/−^ mice. Consistent with our observation, although numerous studies with antioxidant intervention using Oltipraz or vitamin E exhibited a reduction in oxidative stress and fibrosis, none of these treatments prevented hepatocyte injury in acute BDL animals^35^,^36^. Furthermore, NAC promotes GSH synthesis only when GSH is depleted. In mouse BDL model, Yang *et al*. reported that GSH synthase was induced transiently after BDL, but fell to 50% of baseline by 2 weeks^37^. Thus, it is speculated that, in acute phase of BDL, NAC may fail to protect the acute BDL liver of Cygb^−/−^ mice due to increased GSH.

### Loss of Cygb augments liver fibrosis under BDL

One of our findings in this study is the dominant development of fibrosis in Cygb^−/−^ mice upon long-terms of BDL. Previously, we found that the loss of Cygb spontaneously induced the priming of HSCs, which amplified the expression of fibrogenesis-related genes, cytokines, and variety of chemokines^15^. Under BDL, the priming HSCs became fully activated with augmented αSMA expression from acute phase in Cygb^−/−^ mice (Supplementary Fig. S7). Thus, in addition to augmented hepatocyte damage, the prompt activation of HSCs probably contributes to the progression of liver fibrosis in BDL-Cygb^−/−^ mice.

In addition to augmented hepatocyte damage, the priming of HSCs probably contributes to the severe progression of fibrosis in BDL-Cygb^−/−^ mouse liver. In contrast, Cygb transgenic rats exhibited slow progression of fibrosis with ischemia-reperfusion kidney injury^38^. Therefore, the anti-fibrotic function of CYGB could be illuminative. Interestingly, the elevated expression of Cygb in BDL-WT liver (Supplementary Fig. S1) may reflect the important role of Cygb in protective mechanism in cholestasis via scavenging ROS and NO and reducing the activation of HSCs.

In summary, this study demonstrates that Cygb deficiency enhances liver injury and fibrogenesis during cholestasis in mice via the deleterious effects of NO, the impairment of bile canalicular function and the excessive accumulation of toxic bile acids in hepatocytes. This is the first report describing the role of Cygb in cholestatic liver injury, which opens a new window for the understanding the pathophysiology of the disease. The development of drugs that modulate Cygb function and consequently NO metabolism is anticipated for the human cholestatic liver disease, a field of unmet medical needs.

## Methods

### Animal Studies

All mice received humane care according to Guide for the Care and Use of Laboratory Animals, National Institutes of Health. All protocols and experimental procedures were approved by the Institutional Animal Care and Use Committee of Osaka City University and performed in accordance with the guidelines of the National Institutes of Health for the use of animals in research.

Cygb^−/−^ mice (C57BL/6 background) were generated in our laboratory as described previously^14^. C57BL/6 mice (WT) were purchased from SLC (Shizuoka, Japan) and cohoused with Cygb^−/−^ mice at least one week before experiments.

### Bile Duct Ligation Surgery

Cygb^−/−^ and WT male mice from 8 to 11 weeks of age were used. The mice were anaesthetized by an intraperitoneal (i.p.) injection of pentobarbital (Kyoritsu Seiyaku Co., Ltd., Tokyo, Japan) at 60 mg/kg body weight. Then, the peritoneal cavity was opened. The common bile duct was double-ligated by 6-0 suture (Ethicon, San Lorenzo, PR, USA). In sham operation, the bile duct was exposed, but not ligated. The fascia and skin were closed with 5-0 suture (Ethicon). After 24, 48, 72 h or 1, 2, 3 weeks of BDL, mice were scarified. Totally, 12 groups of mice were used with n = 4–8 per BDL group, and n = 3 per sham operated group. For histological examination, 2- to 3-mm-thick liver sections from median and left lobes were fixed in 10% formalin and stained with H&E.

### Treatment of nitric oxide inhibitor and donor

To investigate the role of NO in BDL model, WT or Cygb^−/−^ mice were administered L-NAME (Dojindo, MD, USA) in drinking water at the dose of 0.5 mg/ml for 9 days (n = 5 per each group). At day 7, the mice were performed BDL, and scarified 2 days later.

In NO donor treatment, WT or Cygb^−/−^ mice were received 3 doses of SNP (2 mg/kg body weight) (Millipore Corp., Billerica, MA, USA) or saline by i.p. injection (n = 5 per each group). The first, second and third dose of SNP were injected immediately, 24 h, and 48 h, respectively, after the mice were subjected to BDL. Mice were scarified at 2 h after the last dose.

### NAC treatment

WT or Cygb^−/−^ mice were treated 3 doses of NAC (200 mg/kg body weight) (Sigma-Aldrich, St. Louis, MO, USA) or saline by i.p. injection with the same protocol as SNP treatment (n = 5 per each group).

### Analyses using Histochemistry, Immunohistochemistry, and Immunofluorescence

H&E, SiR-FG, immunohistochemistry, and immunofluorescence were performed as described previously^14^. The primary antibodies that were used for mouse were described in Supplementary Table S1. The area of bile infarcts were calculated by taking microscopic photos of the whole H&E stained liver sections using BZ-X710 microscope (Keyence, Osaka, Japan). Then, BZ-X Analyzer software (Keyence) was used to calculate the percentage of the bile infarct areas divided by total area. To assess apoptosis, TUNEL assay was performed on 5 μm sections with an Apoptosis *in situ* Detection Kit according to the manufacturer’s protocol (MK500; TaKaRa Bio Inc., Shiga, Japan). To access liver fibrosis, sections were stained with SiR-FG. Stained collagen was quantified by taking 10 non-overlapping fields at x200 magnifications per section and using Micro Analysis software version 1.1d (ThermoScientific, FL, USA). To access liver inflammation, neutrophils and macrophages were stained with anti-neutrophil or CD68 antibodies as previously described^15^. Positively immune-stained cells were counted in number by taking 10 fields without overlapping at x400 magnifications per sections. To assess the change of bile canaliculi, liver sections were stained with anti-CD10 antibody. CD10 expression was also quantified following collagen quantification as described above.

### Measurement of AST and ALT and Total Bile Acid Assay

Aspartate transaminase (AST), alanine transaminase (ALT) and total bile acid (TBA) were measured in serum using a commercially available kit (Wako, Osaka, Japan) according to the manufacturer’s protocol.

### Bilirubin assay

Bilirubin in serum was measured by a spectrophotometric assay by using Bilirubin Assay Kit (BioAssay Systems, CA, USA) according to the assay protocol. Briefly, 50 μl serum that was stocked at −80 °C was transferred into 96-well plate (Sigma-Aldrich, Missouri, USA). Next, 200 μl of working reagent was added to the sample wells. The reaction of bilirubin with diazotized sulfanilic acid in working reagent induced a red-colored product. Bilirubin in the unconjugated bilirubin protein complex was split by using caffeine benzoate in working reagent. Then, the reaction mixture was incubated for 10 min at room temperature and read at OD530 nm. Bilirubin content was expressed as mmol per litter.

### Hydroxyproline assay

Hydroxyproline content of the liver was measured by a spectrophotometric assay by using Hydroxyproline Assay Kit (BioVision, CA, USA) as previously described^15^.

### Nitric oxide assay

NO is rapidly oxidized to nitrite (NO_2_^−^) and nitrate (NO_3_^−^) which are used to quantitate NO production. Nitrite and nitrate content of the liver, urine and serum was measured by a spectrophotometric assay by using Nitric Oxide Assay Kit (Abcam, Cambrige, UK) according to the assay protocol. Briefly, two-step process was performed, in which first step converted nitrate to nitrite utilizing nitrate reductase. The second step used Griess reagents to convert nitrite to a deep purple azo compound recorded at 540 nm. The amount of the azochromophore accurately reflects NO amount in samples. NO content was expressed as nmol per litter in serum, nmol per volume of urine 24 h or nmol per gram liver tissue.

Liver tissues that were stocked at −80 °C were homogenized in ice cold assay buffer (100 μl assay buffer for every 10 mg of liver tissue) and then centrifuged at 13,000 g for 3 min at 4 °C. Then, the supernatant was collected. Urine from individual mice was collected over 24 h using silicon wafers that covered the bottom of the mouse cage. Collections occurred at 3 h intervals, including 6:00–9:00, 9:00–12:00, 12:00–15:00, 15:00–18:00, 18:00–21:00, and one 9-h interval from 21:00 until the next day at 6:00 AM. A fresh silicon wafer was placed at each collection time. The total urine volume in a 24 h period was measured and used to calculate the total nmol of nitrate + nitrite in urine per day. Liver lysate, urine and serum were deproteinized before analyses.

### Guanosine 3,5′-cyclic monophosphate assay

Guanosine 3,5′-cyclic monophosphate (cGMP), which is generated via cytoplasmic nitric oxide (NO)-activated guanylate cyclase, is one of the down-stream of NO-dependent cellular signalings. cGMP content of the liver, urine and serum was measured by a spectrophotometric assay by using cGMP assay kit (R&D Systems, Minneapolis, MN, USA), according to the manufacture’s protocol. The liver, urine and serum were prepared similar to NO assay.

### MDA assay

Oxidative stress was assessed by measuring malondialdehyde (MDA), the end products of lipid peroxidation. MDA content of liver and serum was measured by a spectrophotometric assay by using Lipid Peroxidation assay kit (BioVision, Milpitas, CA). Liver tissue was homogenized on ice in MDA lysis buffer, and then centrifuged to remove insoluble material. Thiobarbituric acid (TBA) was added into each sample, incubated at 95 °C for 60 min. MDA in the samples was reacted with TBA to generate the MDA-TBA adduct which were quantified by measuring the absorbance at 532 nm. MDA content was expressed as nmol per ml or nmol per mg liver tissue.

### Caspase 3 activity assay

The caspase 3 activity is one of important hallmarks to assess apoptosis pathway. The caspase 3 activity was measured by using Caspase 3 assay kit (Abcam, Cambrige, UK) according to the assay protocol. Briefly, liver tissue was homogenized on ice in lysis buffer, then, incubated with the substrate DEVD-AFC (AFC: 7-amino-4-trifluoromethyl coumarin) which emits blue light. With the present of activated caspase 3, the substrate DEVD-AFC was cleaved to form free AFC which emits a yellow-green. The cleavage of substrate was quantified by fluorometer. The caspase 3 activity was expressed as RPU per mg protein liver tissue.

### Quantitative Real-Time PCR

The miRNeasy Mini Kit (Qiagen, Valencia, CA, USA) was used to extract total RNA from cells and liver tissues. Then total RNA (1 μg) was used to synthesized cDNAs by a ReverTra Ace qPCR RT Kit (Toyobo, Osaka, Japan) and oligo(dT)12–18 primers accor emits blue light ding to the manufacturer’s instructions. Gene expression was measured by real-time PCR using the cDNAs, SYBR qPCR Mix Reagents (Toyobo), and gene-specific oligonucleotide primers (Supplementary Table S2) with an ABI Prism 7500 Fast Real-Time PCR System (Applied Biosystems, Foster, CA, USA). Glyceraldehyde-3-phosphate dehydrogenase (Gapdh) level was used to normalize the relative abundance of mRNAs.

### Immunoblot Analysis

Protein samples (10 to 40 μg) were subjected to SDS-PAGE and transferred to Immobilon P membranes (Millipore Corp., Billerica, MA). After blocking, membranes were probed with primary antibodies against active + pro caspase 3 (1:1000; Abcam), α SMA (1:1000; Abcam), phosphorylated NF-κB-p65 (1:1000; Cell Signaling, MA, USA), total NF-κB-p65 (1:1000; Delta Biolabs), Cytochrome c (1:1000; Santa Cruz Biotechnology, CA, USA), GAPDH (1:2000; Santa Cruz Biotechnology, CA, USA), CD10 (1:1000; R&D System, MN, USA), iNOS (1:1000; Abcam), HO-1 (1:1000; Assay designs, NY, USA), or CYGB (1:3000; our laboratory). Membranes were then incubated with horseradish peroxidase conjugated secondary antibodies at 1:2000 dilutions. Immunoreactive bands were visualized using the electrochemiluminescence detecting reagent (GE Healthcare UK Ltd, Buckinghamshire), and documented with the Fujifilm Image Reader LAS-3000 (Fujifilm, Tokyo, Japan) coupled with image analysis software (Multi Gauge version 3.1; Fujifilm, Tokyo, Japan).

### Statistical Analysis

All data are expressed as the means ± SD. Two groups were compared using an unpaired Student’s t-test (two tailed). Survival curves were constructed by Kaplan-Meier method and analysed by using Wilcoxon test. P < 0.05 was considered statistically significant.

## Additional Information

**How to cite this article**: Thuy, T. T. V. *et al*. Possible Involvement of Nitric Oxide in Enhanced Liver Injury and Fibrogenesis during Cholestasis in Cytoglobin-deficient Mice. *Sci. Rep.* **7**, 41888; doi: 10.1038/srep41888 (2017).

**Publisher's note:** Springer Nature remains neutral with regard to jurisdictional claims in published maps and institutional affiliations.

## Supplementary Material

Supplementary Information
