# Supplementary material for: Possible Involvement of Nitric Oxide in Enhanced Liver Injury and Fibrogenesis during Cholestasis in Cytoglobin-deficient Mice
Source: Sci Rep. 2017 Feb 3;7:41888. doi: 10.1038/srep41888 (PMC5291093; doi:10.1038/srep41888)
Supplement: Supplementary Information [file srep41888-s1.pdf]

Possible Involvement of Nitric Oxide in Enhanced Liver Injury and Fibrogenesis during Cholestasis in Cytoglobin-deficient Mice

Tuong Thi Van Thuy[1†], Le Thi Thanh Thuy[1†], Katsutoshi Yoshizato[1,2,3], Norifumi Kawada[1*]

[1]Department of Hepatology, Graduate School of Medicine, Osaka City University, Osaka, Japan; [2]Synthetic Biology Laboratory, Graduate School of Medicine, Osaka City University, Osaka, Japan; [3]PhoenixBio Co. Ltd., Hiroshima, Japan

***Corresponding author:** Norifumi Kawada, M.D., Ph.D.; Email: kawadanori@med.osaka-cu.ac.jp

[†]These authors contributed equally to this work.

Supplementary Table S1. Summary of Primary Antibodies Used for Immunohistochemistry or Immunofluorescences

Antigen	Source	Name/clone; catalog no.	Incubation
Active + pro Caspase 3	Abcam	Polyclonal (Rb); ab47131	O/N 4°C, 1:200
CD10	R&D System	Polyclonal (Go); AF1126	O/N 4°C, 1:100
CD11b	Bio-Rad	Monoclonal (Rt) MCA74GA	O/N 4°C 1:100
CD68	Abcam	Polyclonal (Rb); ab125212	O/N 4°C, 1:200
Cytochrome C	Santa Cruz Biotechnology	Monoclonal (Mo); sc-13156	O/N 4°C, 1:100
α-SMA	Abcam	Polyclonal (Go); ab21027	O/N 4°C, 1:100
iNOS	Abcam	Polyclonal (Rb); ab15203	O/N 4°C, 1:100
HO-1	Assay designs	Polyclonal (Rb); SPA-895	O/N 4°C, 1:100
Neutrophil	Abcam	Monoclonal (Rt); ab2557	O/N 4°C, 1:100
Cytoglobin	Our laboratory	Polyclonal (Rb)	O/N 4°C, 1:300

*All antigens were retrieved by autoclaving for 15 min in 0.01 mol/L citrate buffer containing 0.05% Tween 20 (pH 6.0), except for Neutrophil, in which proteinase K (400 mg/mL) in TE buffer (pH 8.0) was used.

Supplementary Table S2. Mouse Primers Used for Quantitative Real-Time PCR

**Primer name**	**Forward primer (5'→3')**	**Reverse primer (5'→3')**
Bsep	CTGCCAAGGATGCTAATGCA	CGATGGCTACCCTTTGCTTCT
Ccl-2	GAGAGCCAGACGGGAGGAAG	TGAATGAGTAGCAGCAGGTGAG
Col 1α1	CCTCCCGCACCCAGTTC	CATCAGCATGTTTGGAGTAGTAAGC
Cxcl-1	TGAGCTGCGCTGTCAGTGCCT	AGAAGCCAGCGTTCACCAGA
Cxcl-2	GAGCTTGAGTGTGACGCCCCCAGG	GTTAGCCTTGCCTTTGTTCAGTATC
Cxcl-5	GCATTTCTGTTGCTGTTCACGCTG	CCTCCTTCTGGTTTTTCAGTTTAGC
Cygb	TGCATGACCCAGACAAGGTA	GGTCACGTGGCTGTAGATGA
Gapdh	TGCACCACCAACTGCTTAG	GGATGCAGGGATGATGTTC
HO-1	GGTGATGGCTTCCTTGTACC	AGTGAGGCCCATACCAGAAG
Icam-1	GTGATGCTCAGGTATCCATCCA	CACAGTTCTCAAAGCACAGCG
Il-6	CCGCTATGAAGTTCCTCTCTGC	ATCCTCTGTGAAGTCTCCTCTCC
iNOS	CCTGGTACGGGCATTGCT	GCTCATGCGGCCTCCTTT
Mdr-2	GCAGCGAGAAACGGAACAG	GGTTGCTGATGCTGCCTAGTT
Mrp-2	GGATGGTGACTGTGGGCTGAT	GGCTGTTCTCCCTTCTCATGG
Mrp-3	TCCCACTTTTCGGAGACAGTAAC	ACTGAGGACCTTGAAGTCTTGGA
Ntcp	ATGACCACCTGCTCCAGCTT	GCCTTTGTAGGGCACCTTGT
Oatp-1	CAGTCTTACGAGTGTGCTCCAGAT	ATGAGGAATACTGCCTCTGAAGTG
Timp-1	ACTCGGACCTGGTCATAAGGGC	TTCCGTGGCAGGCAAGCAAAGT
α-SMA	TCCCTGGAGAAGAGCTACGAACT	AAGCGTTCGTTTCCAATGGT

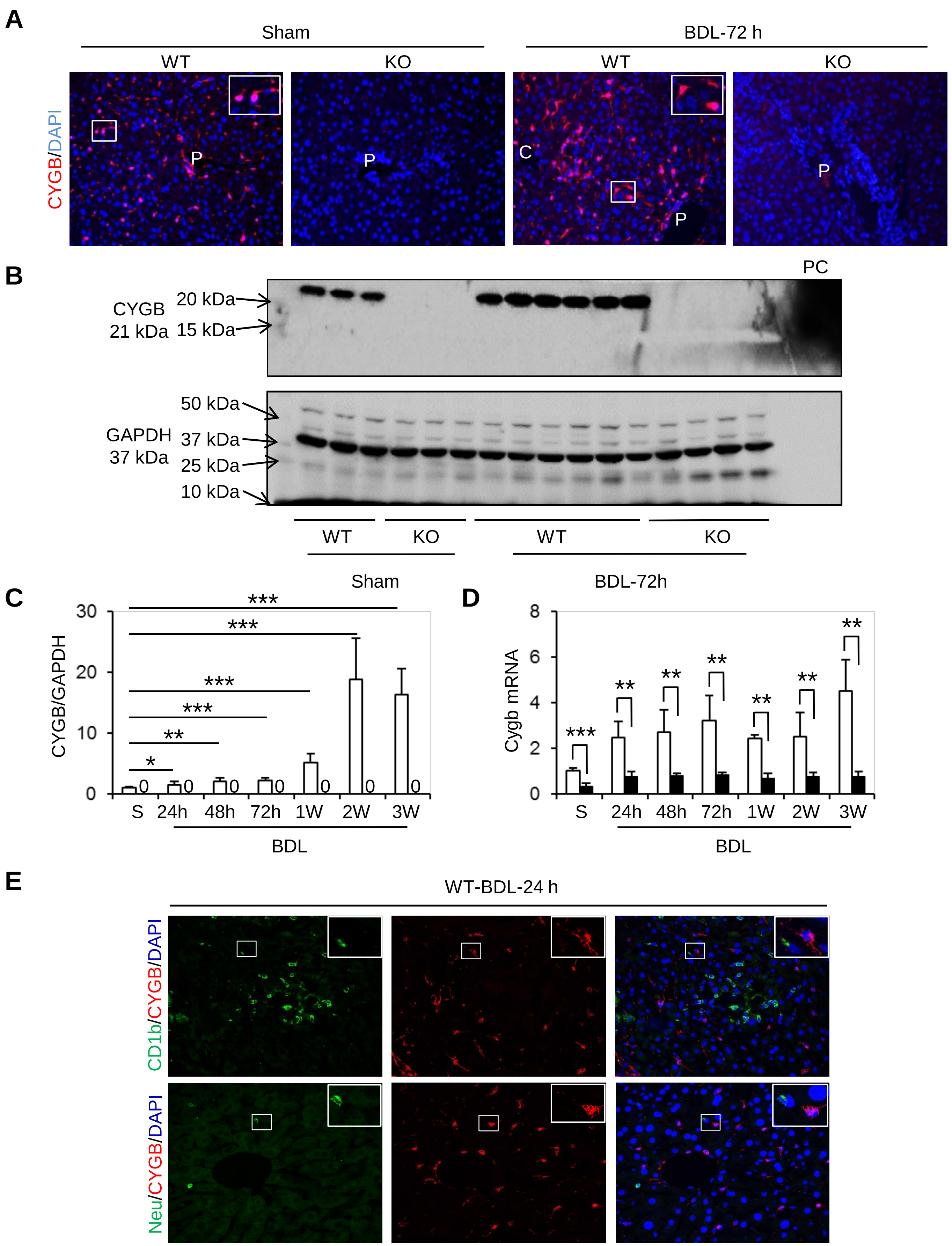
A
Sham
BDL-72 h
WT
KO
WT
KO
CYGB/DAPI
P
P
C
P
P
B
PC
CYGB
21 kDa
20 kDa
15 kDa
GAPDH
37 kDa
50 kDa
37 kDa
25 kDa
10 kDa
WT
KO
WT
KO
Sham
BDL-72h
C
CYGB/GAPDH
30
20
10
0
***
***
***
***
**
*
0
0
0
0
0
0
S
24h
48h
72h
1W
2W
3W
BDL
D
Cygb mRNA
8
6
4
2
0
***
**
**
**
**
**
**
S
24h
48h
72h
1W
2W
3W
BDL
E
WT-BDL-24 h
CD1b/CYGB/DAPI
Neu/CYGB/DAPI

Supplementary Figure S1. Expression of Cytoglobin (Cygb) in bile duct ligation (BDL) mice. (A) Immunofluorescence of CYGB in sham and BDL-72h mice. (B) Immunoblot of CYGB in sham and BDL-72h mice. The human recombinant CYGB protein was used as positive control (PC). GAPDH was used as loading control. All gels were run under the same experimental conditions. Quantitative densitometry (C) and hepatic mRNA level (D) of Cygb at sham (S) mice, acute BDL (24-72h) and chronic BDL (1-3W) mice. (E) Double-immunofluorescence of CYGB and macrophages (CD11b); CYGB and Neutrophil (Neu) in WT-BDL-24h mice. Note that CYGB expression was absent in both macrophages and neutrophils. Open bars, WT; close bars, $Cygb^{-/-}$ (KO). Data represent the mean ± SD. Sham (n = 3), BDL (n = 4-8).* $p < 0.05$, ** $p < 0.01$, *** $p < 0.001$. Original magnifications, x 400, inset, x1200.

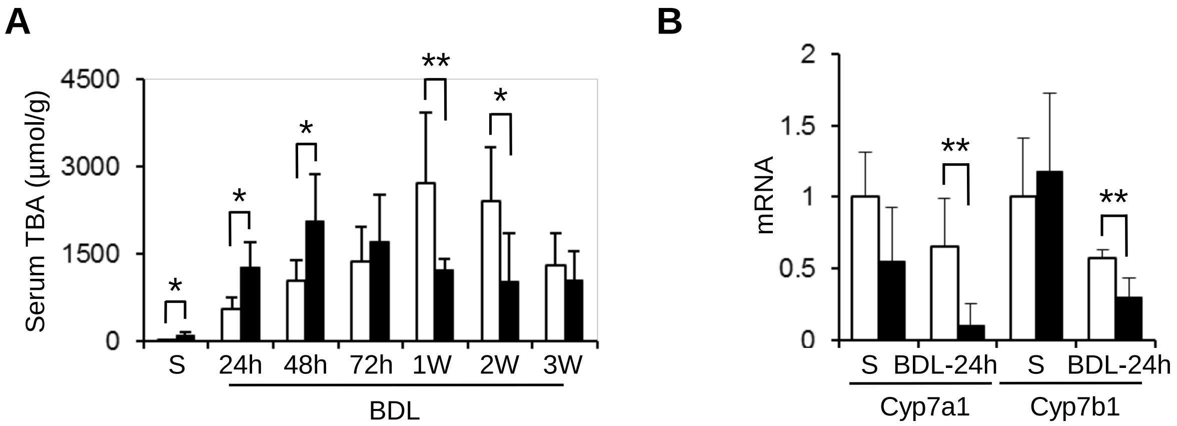

Supplementary Figure S2. Changes in serum total bile acid and bile synthesis in BDL. (A) Serum level of total bile acid (TBA) in sham (S), acute BDL (24-72h), and chronic BDL (1-3W) mice. (B) Hepatic mRNA levels of cytochrome P450 7A1 (Cyp7a1), cytochrome P450 7B1 (Cyp7b1) in sham and acute BDL 24h mice. Sham (n = 3), BDL (n = 4-8). Open bar, WT; closed bar, Cygb$^{-/-}$ (KO). * $p < 0.05$, ** $p < 0.01$.

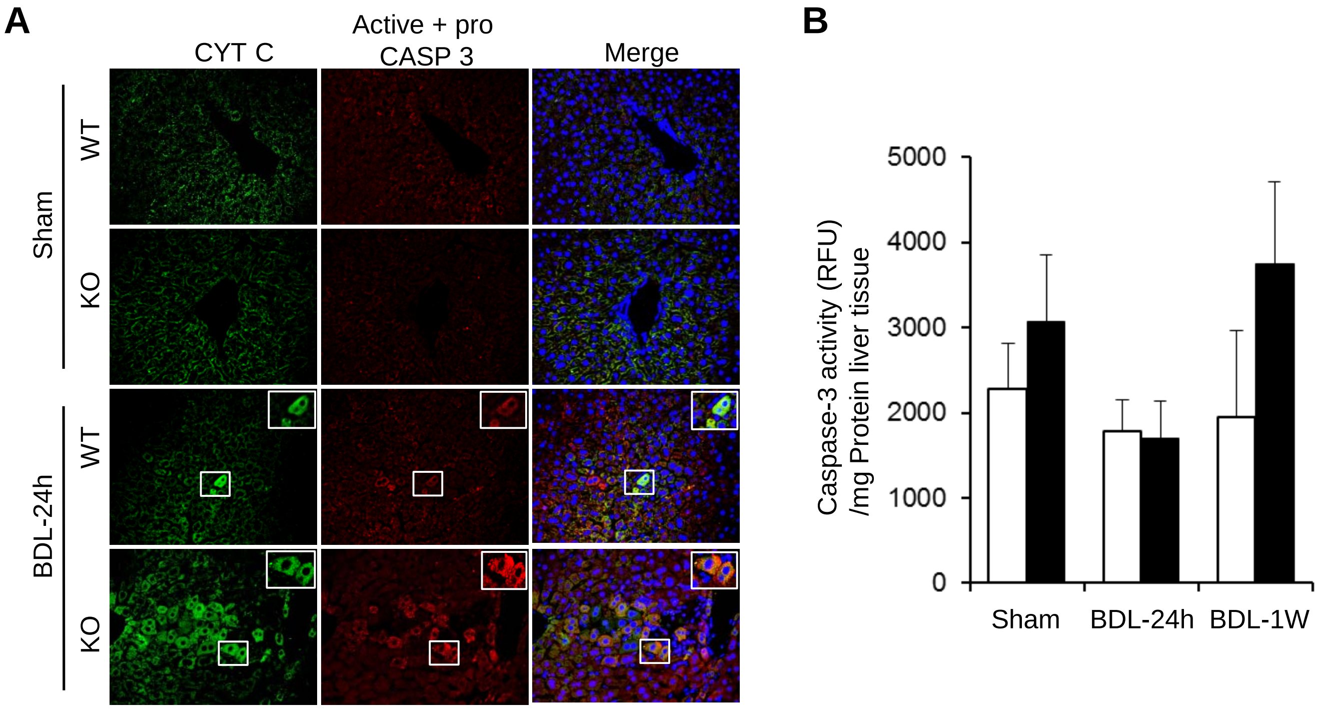

Supplementary Figure S3. Expression of markers of apoptosis in bile duct ligation (BDL) mice. (A) Double immunofluorescence of cytochrome C (CYT C) (green) and active + pro Caspase (CASP) 3 (red) in sham and BDL-24h mice. Insets are higher magnifications of representative areas. (B) Caspase-3 activity in sham and BDL-24h and BDL-1W mice. Open bars, WT; close bars, Cygb$^{-/-}$ (KO). Data represent the mean ± SD. Sham (n = 3), BDL (n = 4-8). Original magnifications, x 400.

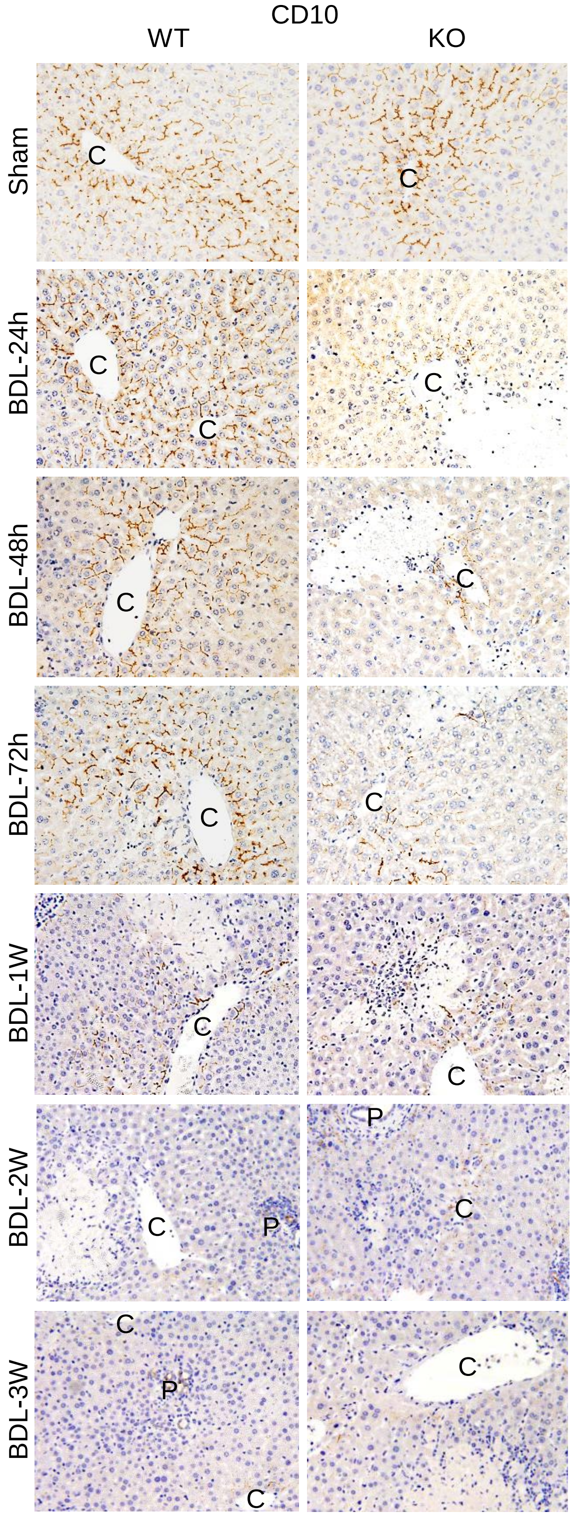
CD10
WT
KO
Sham
BDL-24h
BDL-48h
BDL-72h
BDL-1W
BDL-2W
BDL-3W
C
P

Supplementary Figure S4. Time-dependent changes of CD10 expression following BDL. Immunohistochemistry of CD10. Note that CD10 expression was time-dependently disappeared after BDL in WT but suddenly in Cygb$^{-/-}$ (KO) ones. Sham (n = 3), BDL (n = 4-8). Original magnifications, x 400.

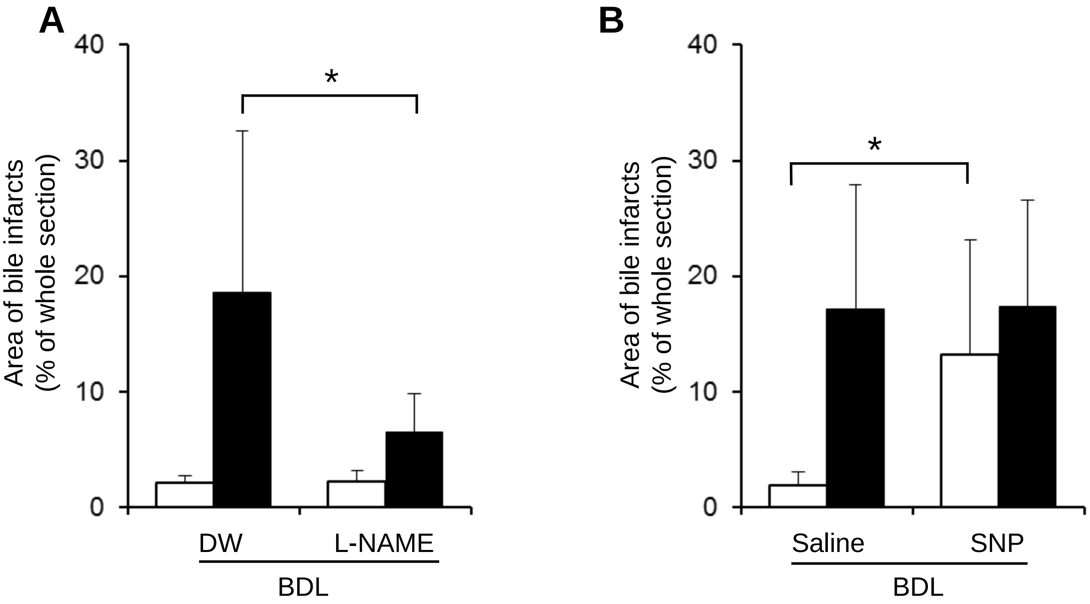

Supplementary Figure S5. Effect of NO inhibitor and NO donor in liver injury in BDL 48 hour. (A) Percentage of area of bile infarcts in mice with (A) L-NAME treatment and control (drinking water (DW)) or (B) SNP treatment and control (saline) under BDL 48 hour. (n = 5). Open bar, WT; closed bar, $Cygb^{-/-}$ (KO). * $p < 0.05$.

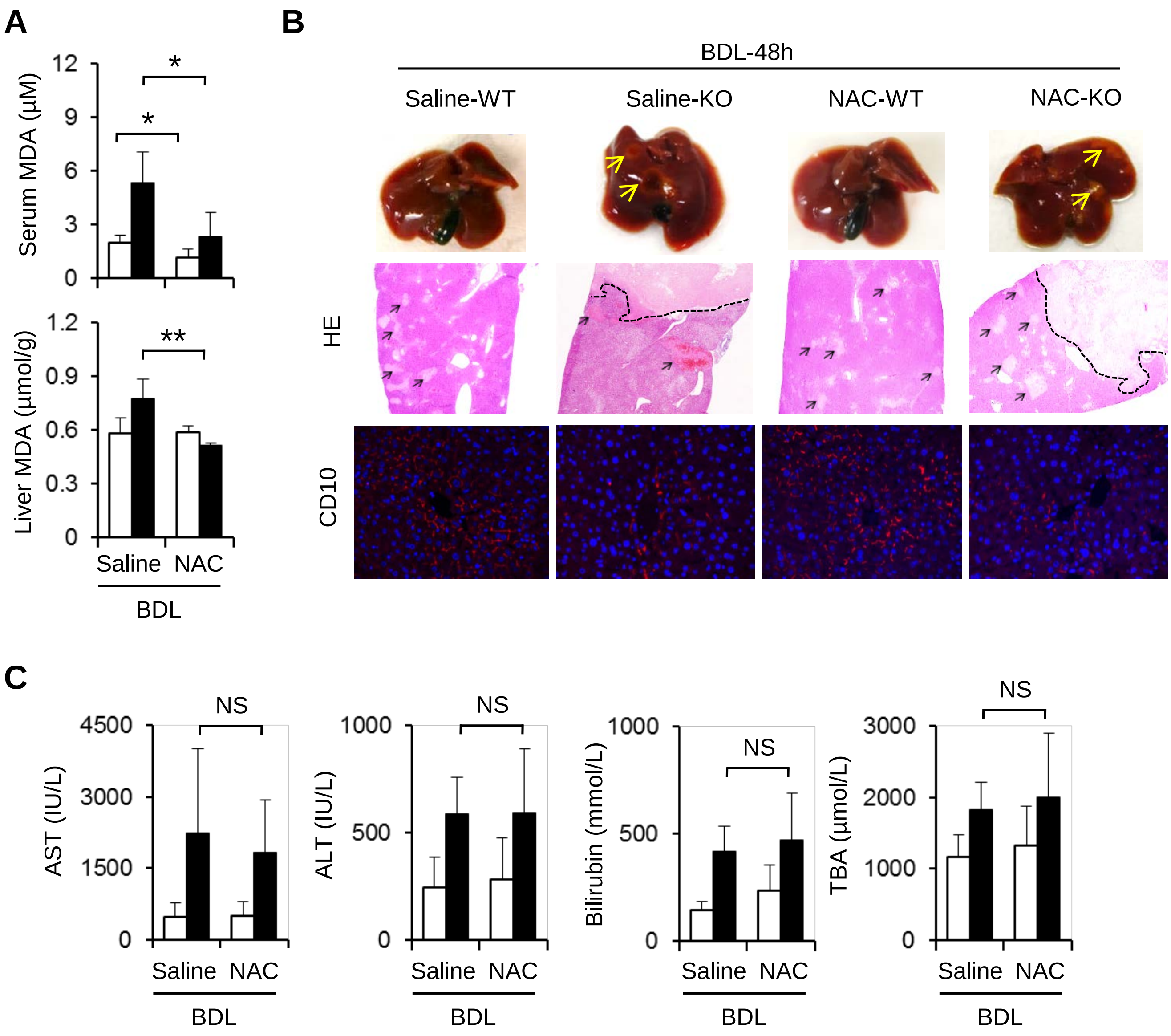

Supplementary Figure S6. Effect of N-Acetylcysteine in liver injury after BDL. (A) Malondialdehyde (MDA) content in serum and liver under saline or N-acetylcysteine (NAC) administration. (B) Representative macroscopic images, microscopic liver sections stained with hematoxylin and eosin (H&E) and immunofluorescence staining for CD10 in BDL-48h treated mice together with saline or NAC administration. (C) Serum aspartate transaminase (AST), alanine transaminase (ALT), total bilirubin, and total bile acid (TBA). Open bar, WT; closed bar, Cygb$^{-/-}$ (KO). Data represent the mean ± SD. n = 5. * $p < 0.05$, ** $p < 0.01$. Original magnifications, x 40 (H&E) and x 400 (CD10).

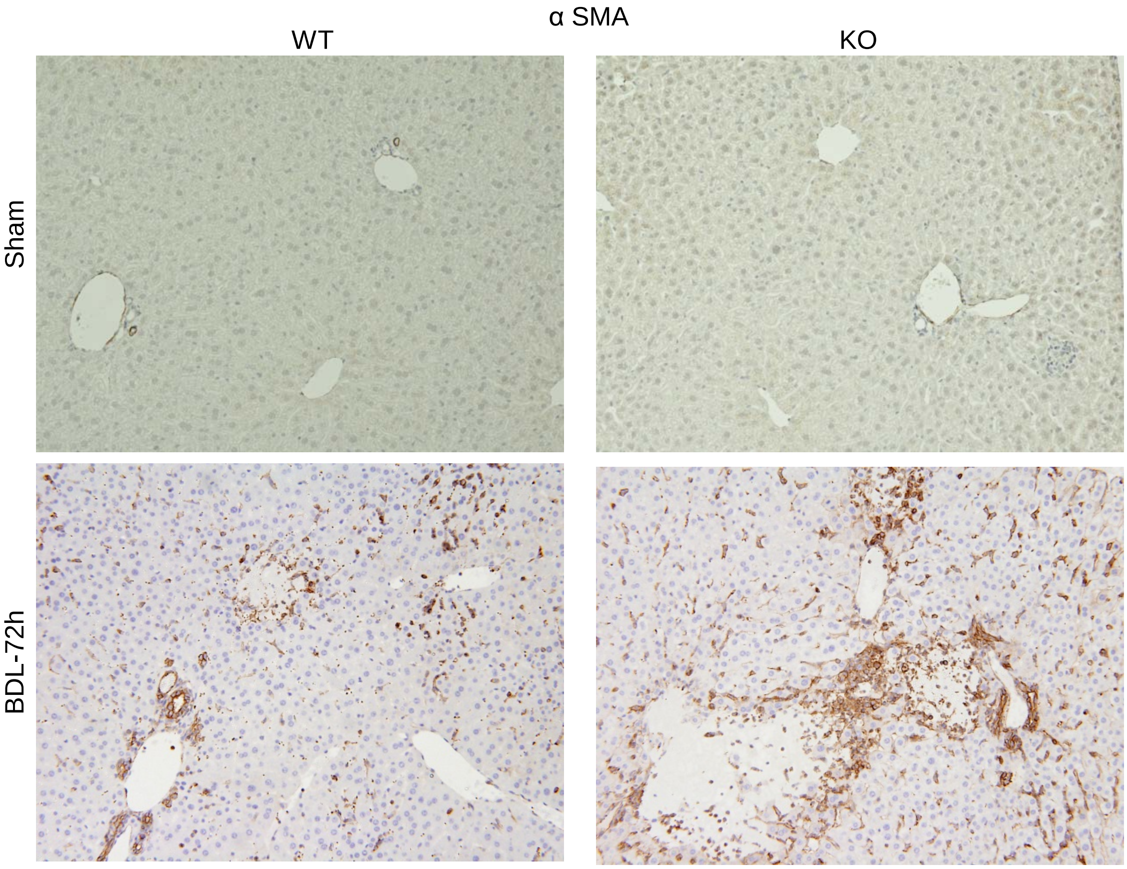

Supplementary Figure S7. Activated hepatic stellate cells (HSCs) in BDL-72h. Immunohistochemistry of α-SMA. Original magnifications, x 200.

Figure 2E

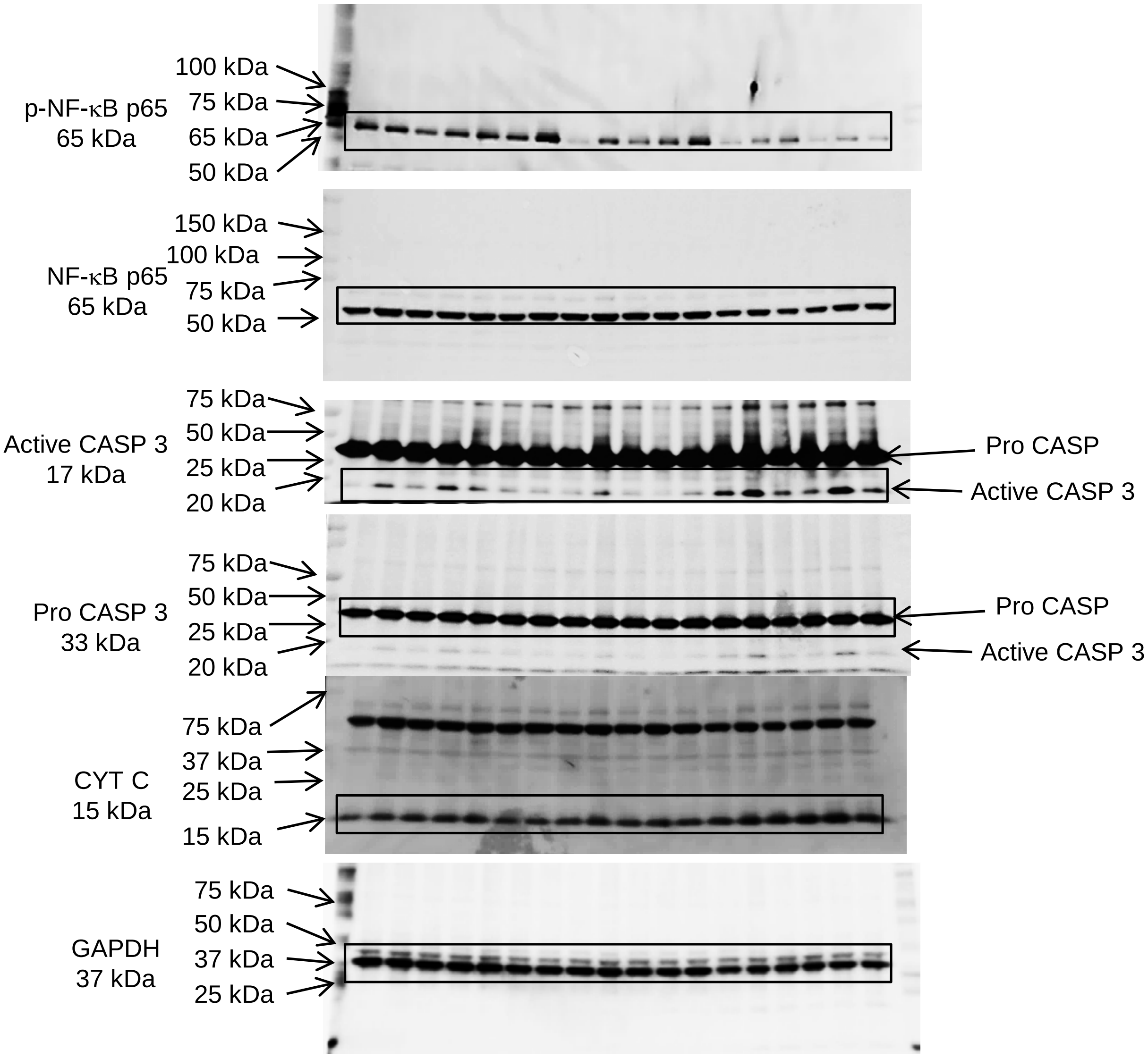

Supplementary Figure S8: Uncropped scans of Western blots shown in Figure 2.

Original images of Western blots. Boxes areas indicated the cropped regions.

Figure 3C

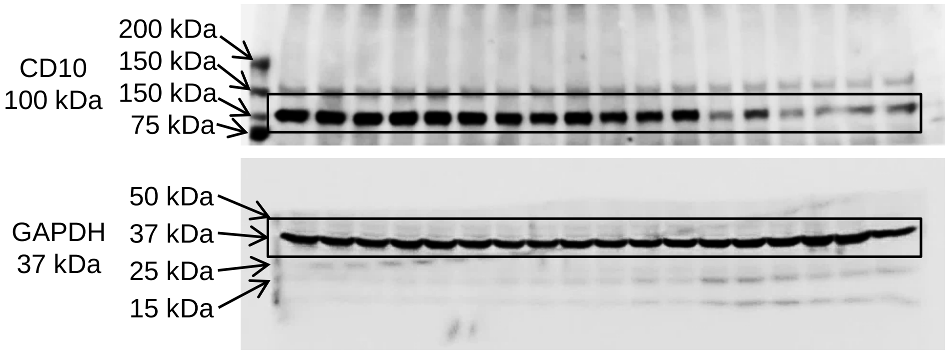

Supplementary Figure S9: Uncropped scans of Western blots shown in Figure 3.

Original images of Western blots. Boxes areas indicated the cropped regions.

Figure 4C

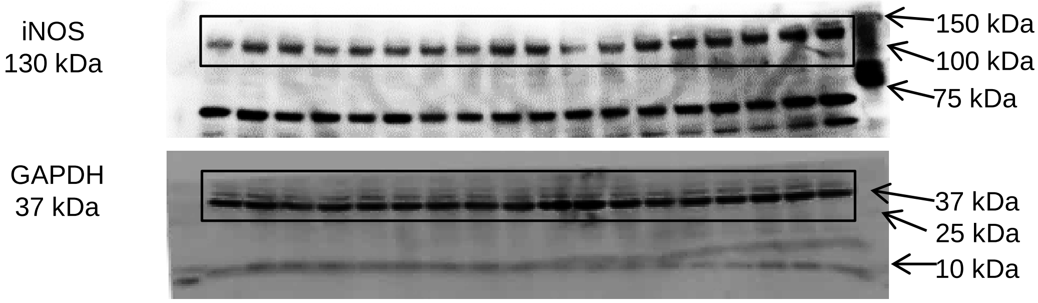

Figure 4E

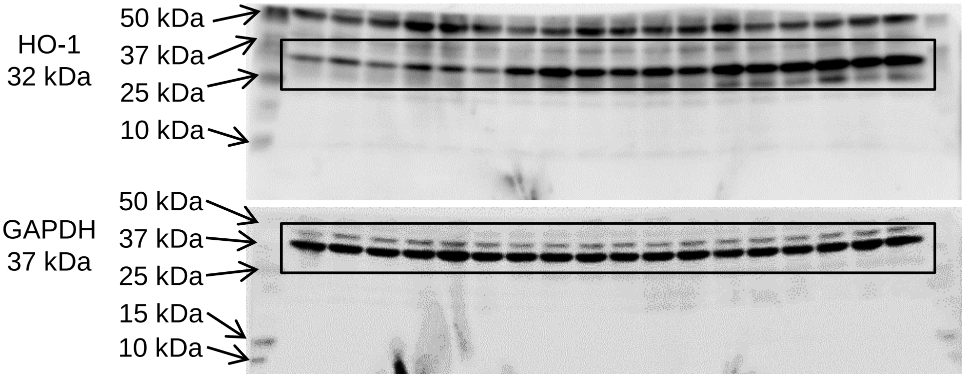

Supplementary Figure S10: Uncropped scans of Western blots shown in Figure 4.

Original images of Western blots. Boxes areas indicated the cropped regions.

Figure 5D

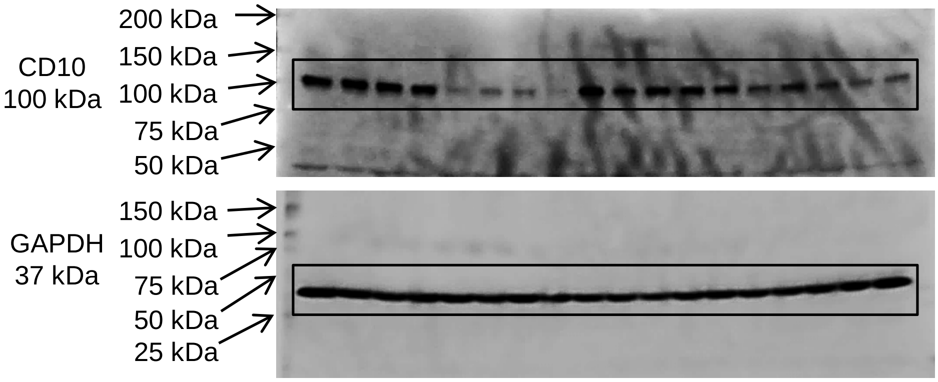

Supplementary Figure S11: Uncropped scans of Western blots shown in Figure 5.

Original images of Western blots. Boxes areas indicated the cropped regions.

Figure 6D

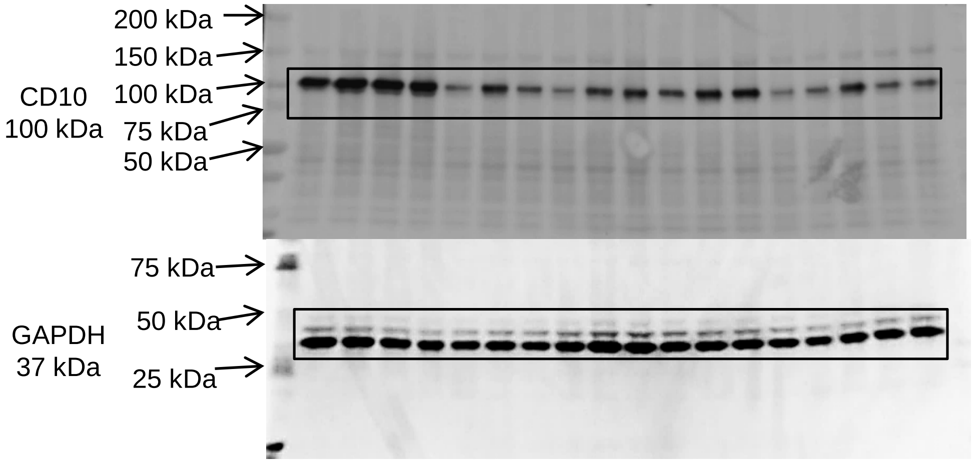

Supplementary Figure S12: Uncropped scans of Western blots shown in Figure 6.

Original images of Western blots. Boxes areas indicated the cropped regions.

Figure 7C

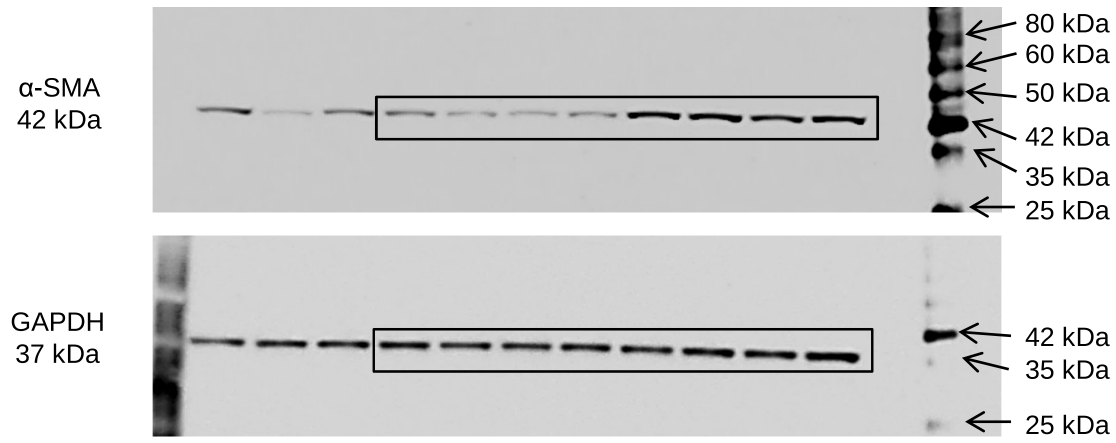

Supplementary Figure S13: Uncropped scans of Western blots shown in Figure 7.

Original images of Western blots. Boxes areas indicated the cropped regions.
